# A compact regulatory RNA element in mouse Hsp70 mRNA

**DOI:** 10.1093/narmme/ugae002

**Published:** 2024-01-29

**Authors:** Wenshuai Wang, Fei Liu, Maria Vera Ugalde, Anna Marie Pyle

**Affiliations:** Department of Molecular, Cellular and Developmental Biology, Yale University, New Haven, CT 06511, USA; Howard Hughes Medical Institute, Yale University, New Haven, CT 06520, USA; Department of Molecular, Cellular and Developmental Biology, Yale University, New Haven, CT 06511, USA; Howard Hughes Medical Institute, Yale University, New Haven, CT 06520, USA; Department of Biochemistry. McGill University, Montreal, Quebec H3G 1Y6, Canada; Department of Molecular, Cellular and Developmental Biology, Yale University, New Haven, CT 06511, USA; Howard Hughes Medical Institute, Yale University, New Haven, CT 06520, USA

## Abstract

Hsp70 (70 kDa heat shock protein) performs molecular chaperone functions by assisting the folding of newly synthesized and misfolded proteins, thereby counteracting various cell stresses and preventing multiple diseases, including neurodegenerative disorders and cancers. It is well established that, immediately after heat shock, Hsp70 gene expression is mediated by a canonical mechanism of cap-dependent translation. However, the molecular mechanism of Hsp70 expression during heat shock remains elusive. Intriguingly, the 5′ end of Hsp70 messenger RNA (mRNA) appears to form a compact structure with the potential to regulate protein expression in a cap-independent manner. Here, we determined the minimal length of the mHsp70 5′-terminal mRNA sequence that is required for RNA folding into a highly compact structure. This span of this RNA element was mapped and the secondary structure characterized by chemical probing, resulting in a secondary structural model that includes multiple stable stems, including one containing the canonical start codon. All of these components, including a short stretch of the 5′ open reading frame (ORF), were shown to be vital for RNA folding. This work provides a structural basis for future investigations on the role of translational regulatory structures in the 5′ untranslated region and ORF sequences of Hsp70 during heat shock.

## Graphical Abstract

**Graphical Abstract:**
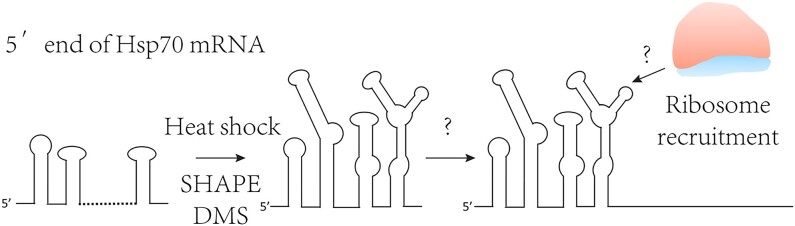

## Introduction

Hsp70 (70 kDa heat shock protein) is an indispensable and highly conserved protein chaperone, which functions by stabilizing newly synthesized proteins to ensure correct protein folding and refolding during cell stress, thereby modulating metabolic and immune responses and promoting cell survival (1–5). The dysregulation of molecular chaperone Hsp70 has been associated with multiple diseases (5–7). Therefore, understanding the mechanisms of Hsp70 translation regulation and gene expression is critical for fully exploiting the potential of Hsp70 in the development of novel therapeutic strategies.

Years of study have established that, upon stresses such as heat shock, the resulting misfolded proteins interact with an HSP (heat shock protein), thereby releasing the transcription factor HSF1 (heat shock factor 1), which is transported into the nucleus, where it initiates transcription of other HSPs (8,9). As a result, HSPs such as Hsp70 in the cytosol become dramatically upregulated, thereby efficiently activating molecular chaperone functions. However, translational regulatory mechanisms during heat shock stimulus are not well understood, especially when well-characterized cap-dependent translation mechanisms of Hsp70 are prohibited (10). This strongly suggests that Hsp70 can be expressed in multiple ways, including some form of cap-independent translation mechanism that can occur under stress conditions. It is well known that certain RNA structures such as IRES (internal ribosome entry site) motifs, located in the 5′ end of specific types of messenger RNAs (mRNAs), can help recruit ribosomes, where they initiate cap-independent translation (10). As the 5′ untranslated region (UTR) of Hsp70 mRNA has been reported to play a positive role during cap-independent Hsp70 translation (11–16), we hypothesized that 5′-terminal regions of Hsp70 mRNA may fold into a compact structure that assists in cap-independent Hsp70 translation during heat shock stress.

To learn more about the architecture of the Hsp70 5′ end, we examined the influence of mHsp70 mRNA truncations on RNA folding, and we then probed the RNA secondary structure, identifying unique features that contribute to formation of a compact structure. To our surprise, we found that the minimal construct (UTR26) capable of folding to a compact structure is composed of the 5′ UTR and part of the open reading frame (ORF) sequence from the mHsp70 mRNA. UTR26 contains a network of stable RNA stems, including a stem that contains the start codon. These motifs all contribute to the stability of RNA folding, thereby providing a structural basis for understanding the function of a putative regulatory RNA structure in Hsp70 translation during heat shock.

## Materials and methods

### Plasmid expression constructs

A series of T7 RNA transcription constructs containing variations in the 5′ end sequence of mouse Hsp70 mRNA were cloned into the pBluescript vector (Agilent) using Q5 Site-Directed Mutagenesis Kit (NEB). The resulting plasmids were then overexpressed, cleaved to the appropriate length with restriction enzyme BamHI (NEB) and then used for *in vitro* transcription of RNA constructs used in this study. Specifically, plasmids pUTR1 [1–231, numbering is the span from the mRNA 5′ end (position 1) to the position within the gene (position 231 in this case)], pUTR2 (1–261), pUTR4 (1–285), pUTR7 (1–331), pUTR9 (1–381), pUTR10 (1–315), pUTR21 (1–372), pUTR22 (1–363), pUTR23 (1–354), pUTR24 (1–345), pUTR25 (1–336) and pUTR26 (1–327) encode the corresponding RNAs used in this study. The unzipping and compensatory RNA mutants are encoded by plasmids pM9a-2 (H1 unzipping mutant 1a) and pM9a-2C (H1 compensatory mutant 1b), pM9e (H1 unzipping mutant 2a) and pM9eC (H1 compensatory mutant 2b), pM7f (H4 unzipping mutant 1a) and pM7fC (H4 compensatory mutant 1b), pM7h (H4 unzipping mutant 2a) and pM7hC (H4 compensatory mutant 2b), pM1-2 (H6 unzipping mutant 1a) and pM1-2C-AUG (H6 compensatory mutant 1b), and pM5-2 (H6 unzipping mutant 2a) and pM5-2C-AUG (H6 compensatory mutant 2b).

### *In vitro* transcription and denatured purification of RNA

Following linearization of each expression construct, Hsp70 RNAs (Supplementary Table S1) were *in vitro* transcribed using T7 RNA polymerase that was expressed and prepared as described previously (17). Specifically, a 300 μl transcription solution contained 30 μg of linearized plasmid, 40 mM Tris–HCl (pH 8.0), 15 mM MgCl_2_, 10 mM DTT, 2 mM spermidine, 0.01% Triton X-100, 4 mM of each NTP (ATP, UTP, CTP), 5 mM of GTP, 80 U of RNaseOUT™ Recombinant Ribonuclease Inhibitor (Thermo Fisher) and 0.1 mg/ml of purified T7 RNA polymerase (18). A 40 μl transcription solution for body-labeled Hsp70 RNAs was prepared similarly as above, with modifications such as the use of 2 μg of linearized plasmids, and 4 mM final concentration of each NTP (ATP, CTP, GTP), 1 mM UTP and 50 μCi α-UT^32^P. Transcription reactions were incubated for 2 h at 37°C and the resulting RNAs were purified by denaturing gel electrophoresis using a 5% denaturing polyacrylamide gel containing 7 M urea.

### Native folding test

To monitor RNA folding, 10 nM body-labeled denatured RNA or 1 μM unlabeled denatured RNA (see above) was incubated in a folding buffer containing 20 mM HEPES-K (pH 7.5), 0.1 mM EDTA-Na (pH 8.0), 50 mM KCl and varying MgCl_2_ concentrations, at a final volume of 10 μl. After a 40-min incubation at 43°C, the RNA folding state was monitored by gel-shift separation of the RNA using native 5% polyacrylamide gel electrophoresis.

### Chemical probing

Optimized RNA construct UTR26 was folded as described above, and then purified by size-exclusion chromatography using an S200 10/300 column (GE) equilibrated in a buffer containing 20 mM potassium cacodylate (pH 7.0), 0.1 mM EDTA-Na (pH 8.0), 50 mM KCl and 5 mM MgCl_2_. Two different types of chemical probing were conducted.

#### Selective 2′-hydroxyl acylation analyzed by primer extension

SHAPE (selective 2′-hydroxyl acylation analyzed by primer extension) reactions were performed in a 270 μl volume, preparing 5 pmol of folded UTR26 in 243 μl of a buffer containing 20 mM potassium cacodylate (pH 7.0), 0.1 mM EDTA-Na (pH 8.0), 50 mM KCl and 5 mM MgCl_2_, followed by incubation with 27 μl of freshly prepared 50 mM 1M7 (1-methyl-7-nitroisatoic anhydride) (19) in anhydrous dimethyl sulfoxide (DMSO), at 37°C for 10 min. The untreated, negative control RNA was combined with pure anhydrous DMSO (10%). Reactions were quenched with 20 μl of quench buffer containing 1.5 M NaOAc (pH 5.2), 0.25 M EDTA-Na (pH 8.0) and 0.5 mg/ml glycogen (Thermo Fisher). Following precipitation with 70% ethanol, the resulting RNA sample was then dried and dissolved in ddH_2_O and cleaned with an RNA Clean & Concentrator Kit (Zymo Research). Reverse transcription was carried out as previously described (20). In brief, 0.1 μM JOE-conjugated reverse primer (5′-JOE-GATGATCTCCACCTTGCCGT-3′; JOE = 6-carboxy-4′,5′-dichloro-2′,7′-dimethoxyfluorescein) was extended on 1 pmol template RNA using SuperScript III Reverse Transcriptase (Thermo Fisher). Quenching of this reaction and DNA precipitation were performed as described above.

#### DMS probing

Dimethyl sulfate (DMS) treatment reactions were performed in a 270 μl volume, preparing 5 pmol of folded UTR26 in 243 μl of a buffer containing 20 mM potassium cacodylate (pH 7.0), 0.1 mM EDTA-Na, 50 mM KCl and 5 mM MgCl_2_, followed by incubation with 27 μl of freshly prepared 0.77% (v/v) DMS (Sigma–Aldrich) in 100% ethanol, at 37°C for 10 min. The negative control RNA was incubated in the same way but in the absence of DMS (10% EtOH only). Reactions were quenched with 50 μl of 5% (v/v) 2-mercaptoethanol in 100% ethanol. Sample preparation and reverse transcription were carried as described above for SHAPE.

### Sequencing and structure mapping by capillary electrophoresis

The synthesis of fluorescent primers, preparation of sequencing ladders and capillary electrophoresis methods were conducted as previously described (20).

#### Data processing, normalization and error assessment

Both SHAPE and DMS data were processed with QuShape as previously described (20,21). The SHAPE reactivity profiles for each nucleotide were obtained by subtracting the peak areas of untreated control samples (−) from the peak areas of chemically treated samples (+), followed by normalization (21). The DMS reactivity of adenosines and cytosines was normalized separately due to the intrinsic different reaction rates of DMS (22). All the SHAPE and DMS probing was reproduced seven and five times, respectively.

#### Secondary structure determination

The software program RNAstructure (https://rna.urmc.rochester.edu/RNAstructure.html) was used to predict secondary structures of UTR26 using the SHAPE reactivities as pseudo-energy constraints (23). The resulting predicted structures were manually compared with the DMS data. Secondary structures were drawn with VARNA (http://varna-gui.software.informer.com/).

#### Shannon entropy calculation

The Shannon entropy was calculated as previously described (24,25). The Shannon entropy, ranging from 0 to 1, serves as a quantification of uncertainty regarding the identity of an individual RNA secondary structure, distinct from an ensemble of RNA structures. Elevated Shannon entropy values signify structurally dynamic regions, suggesting variability and flexibility in the RNA structure. Conversely, lower entropy values indicate more stable structural elements characterized by discrete and specific secondary structures (26).

## Results

### A compact structure at the 5′ end of mHsp70 mRNA

**Figure 1.**
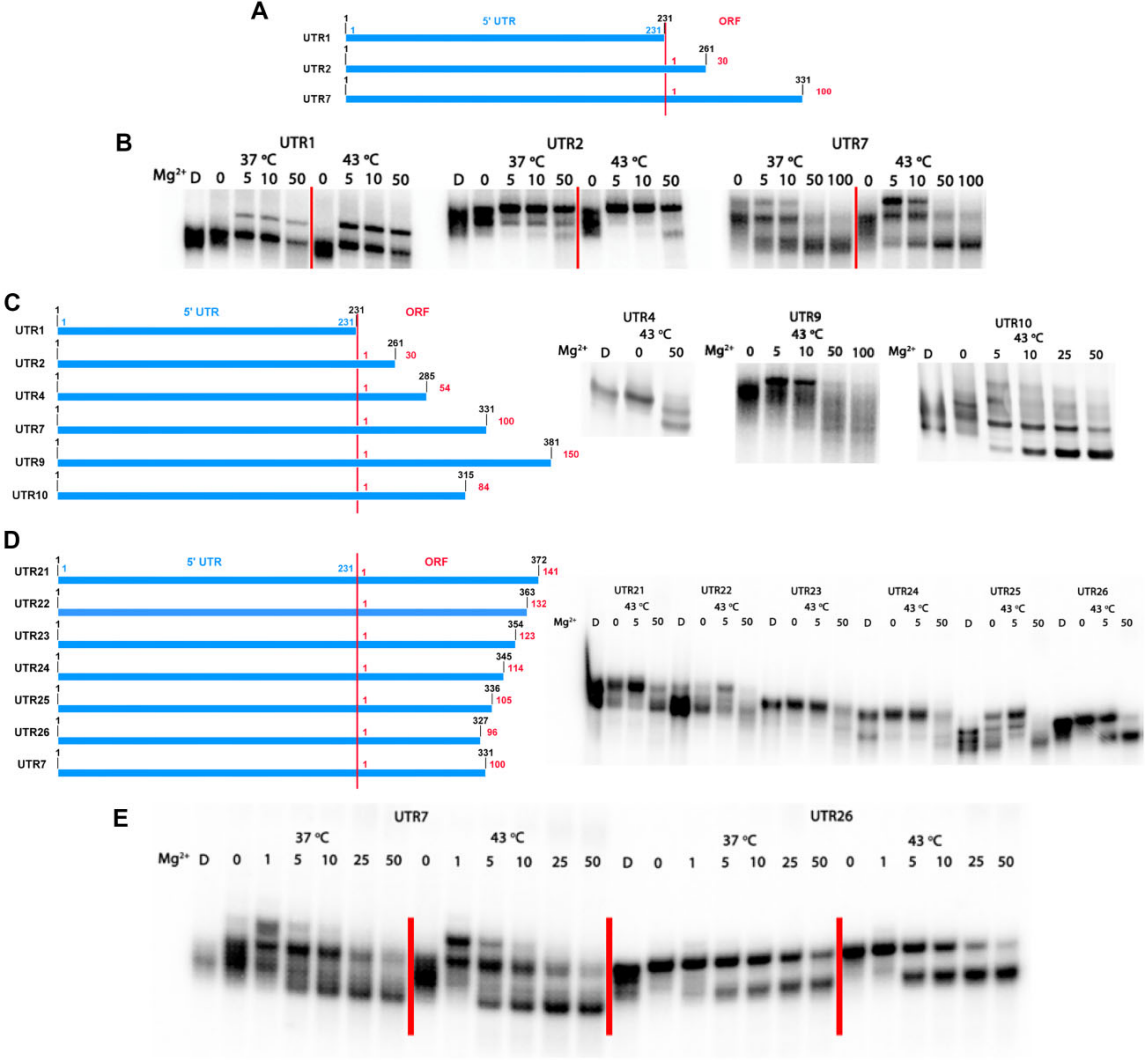
The 5′ end of Hsp70 mRNA folds to a compact structure. (**A**) Schematic representation of different RNA constructs containing 5′ end of Hsp70 mRNA. The truncations, 5′ UTR and ORF sequences are numbered. (**B**) Folding results of RNA constructs described above in native gel at 37 and 43°C. The denatured RNA sample is labeled as ‘D’. The Mg^2+^ concentrations are shown as 0, 5, 10 and 50 mM. Species migrating more slowly in the Mg^2+^ lanes of UTR1 are not compact, and may indicate aggregation. Species migrating more rapidly in the Mg^2+^ lanes of UTR7 represent compact, folded species. (**C**, **D**) Schematic representation of different RNA constructs for determining the minimal folding truncation, and corresponding folding results of these truncations. (**E**) Folding results of two best truncations (UTR7, UTR26) showing well-defined folded RNA band. The body-labeled (α-UT^32^P) RNAs were used in the folding assay.

We hypothesized that the 5′ end of mHsp70 mRNA folds to a compact structure, potentially acting as a thermosensor to mediate cap-independent translation of Hsp70 during heat shock. To test this hypothesis, the 5′ UTR sequence (designated UTR1) of mHsp70 mRNA was *in vitro* transcribed and the different RNA species were separated by native gel electrophoresis to assess the relative level of compaction under normal (37°C) and heat shock (43°C) conditions. In general, folded, more compact RNAs migrate faster in a native gel than unfolded RNAs (27). Surprisingly, we found that the 5′ UTR in isolation (RNA construct UTR1) cannot fold into a compact structure *in vitro*, as indicated by the lack of a compact RNA band for the UTR-only construct (Figure 1A and B). In contrast, when 100 nucleotides of the 5′-terminal ORF sequence are appended downstream from the UTR (resulting in construct UTR7), the RNA folds into a compact structure under both normal and heat shock conditions (Figure 1A and B), suggesting an important role for ORF sequences in structural function of the 5′-terminal region of mHsp70.

To identify the ORF sequences that contributed to 5′-terminal mHsp70 folding, we created a set of new constructs in which 30-nt, 54-nt, 84-nt and 150-nt ORF extensions were sequentially added downstream of the 5′ UTR (UTR2, UTR4, UTR10 and UTR9). However, none of these RNA constructs folded with greater compaction than UTR7 (Figure 1A–C), confirming the unique contribution of the 100-nt ORF sequences contained within UTR7. To explore the minimal sequence required for the folding process, we fine-tuned the ORF length based on UTR7 and tested folding of the resultant constructs. Intriguingly, when compared with UTR7, the removal of four ORF nucleotides (resulting in UTR26) resulted in a construct with better overall compaction, and in this case, apparent temperature-dependent folding, as illustrated by the more homogeneous and temperature-sensitive compact bands of UTR26 in the native gel (Figure 1D and E). All other RNA truncations failed to enhance folding (Figure 1D), suggesting that the downstream ORF region of UTR7 and UTR26 is critical for the compaction process and that it may contribute important structural elements. These findings suggest that the 5′ UTR of mHsp70, together with a segment of the downstream ORF, forms an architecturally compact RNA motif, the structure of which is regulated by temperature.

### Determination of the secondary structure

**Figure 2.**
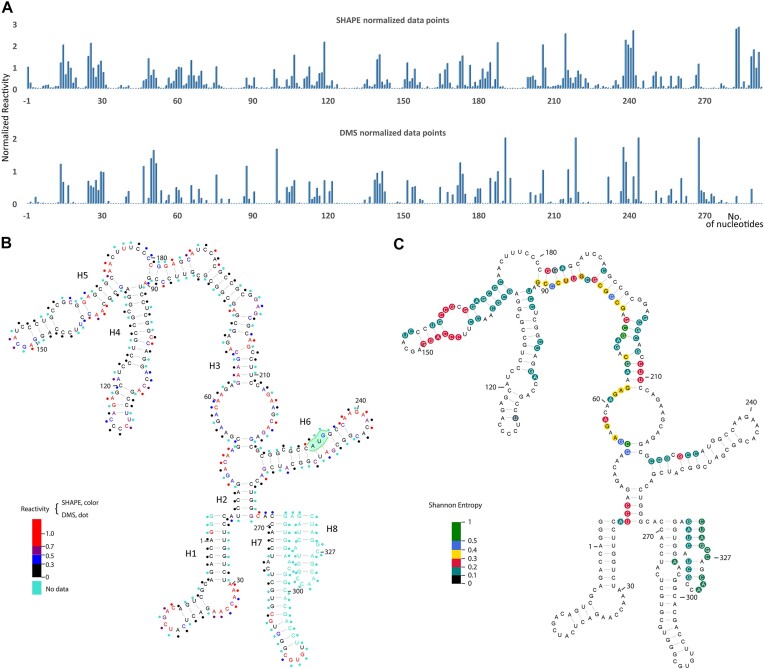
Predicted secondary structure of 5′ end of Hsp70 mRNA. (**A**) Representative normalized SHAPE/DMS reactivity profiles of each nucleotide position. (**B**) SHAPE reactivities are presented by colored nucleotides, while DMS reactivities are rendered as colored dots next to the nucleotides. Highly reactive nucleotides (>0.5), low reactive nucleotides (<0.5) and nucleotides with no data are indicated in the legend. The canonical start codon, AUG, is highlighted. (**C**) Nucleotides with high Shannon entropy values (>0.4), medium Shannon entropy values (0.2–0.4), and low Shannon entropy values (<0.2) are indicated in the legend.

Having shown that the minimal construct UTR26 folds into a compact structure, we utilized SHAPE and DMS probing to characterize its secondary structure. Having isolated this well-folded RNA using a size-exclusion column (see the ‘Materials and methods’ section and Supplementary Figure S1), we then probed the secondary structure of UTR26 RNA at 43°C using SHAPE and DMS probing (Supplementary Figure S2). SHAPE reagents such as 1M7 selectively acetylate the 2′-hydroxyl group of RNA nucleotides with flexible backbones, and DMS selectively methylates the heterocyclic nitrogen atoms on unpaired adenines and cytosines (19,22,28,29). A robust secondary structure is established by the agreement between these two canonical chemical probing approaches (20). We monitored SHAPE reactivity at single-nucleotide resolution and used normalized SHAPE reactivities as pseudo-free energy constraints to restrain the secondary structure predication by minimum free energy (Figure 2A and B). To evaluate the SHAPE-restrained secondary structure model, we then aligned the DMS reactivity to the model, observing that the DMS reactivity data matched well with the SHAPE reactivity (Figure 2A and B), suggesting good agreement between the two methods. The resulting predicted model is composed of eight stem loops (H1–H8, Figure 2B), suggesting that UTR26 RNA has a discrete architecture and that it can form a compact motif containing about 55% of base-paired nucleotides (Figure 2B). Interestingly, both termini of UTR26 fold into stable stem-loop elements, emphasizing their importance during the folding process. More importantly, the canonical start codon is located in stem H6, suggesting a potential role for this stem in regulated translation of Hsp70 during heat shock.

### Mutational analysis and testing of the compact Hsp70 5′-terminal structure

Having obtained a preliminary secondary structure model of UTR26 RNA, we next evaluated this model by testing a series of unzipping and compensatory mutants. The unzipping mutants were designed to disrupt folding to the compact state, while the corresponding compensatory mutants were designed to restore disrupted folding capacity. To determine the stem loops to be unzipped, we evaluated the potential local structural heterogeneity by calculating the Shannon entropy of each nucleotide based on the SHAPE-directed base-pair probabilities (Figure 2C) (25). The Shannon entropy range from 0 to 1 is used a measure of uncertainty in the identity of an individual RNA secondary structure (as opposed to an ensemble of RNA structures). High Shannon entropy indicates structurally dynamic regions, while low entropy suggests more stable structural elements that have a discrete, specific secondary structure (25,26,30). The average Shannon entropy was about 0.1, indicating a high level of structural certainty in the predicted UTR26 conformation, and a low probability of alternative conformers. Among the eight stem loops, nucleotides of stem loops H1, H4 and H6 had the lowest Shannon entropy (<0.2), suggesting that these three stem loops are more stable (Figure 2C).

**Figure 3.**
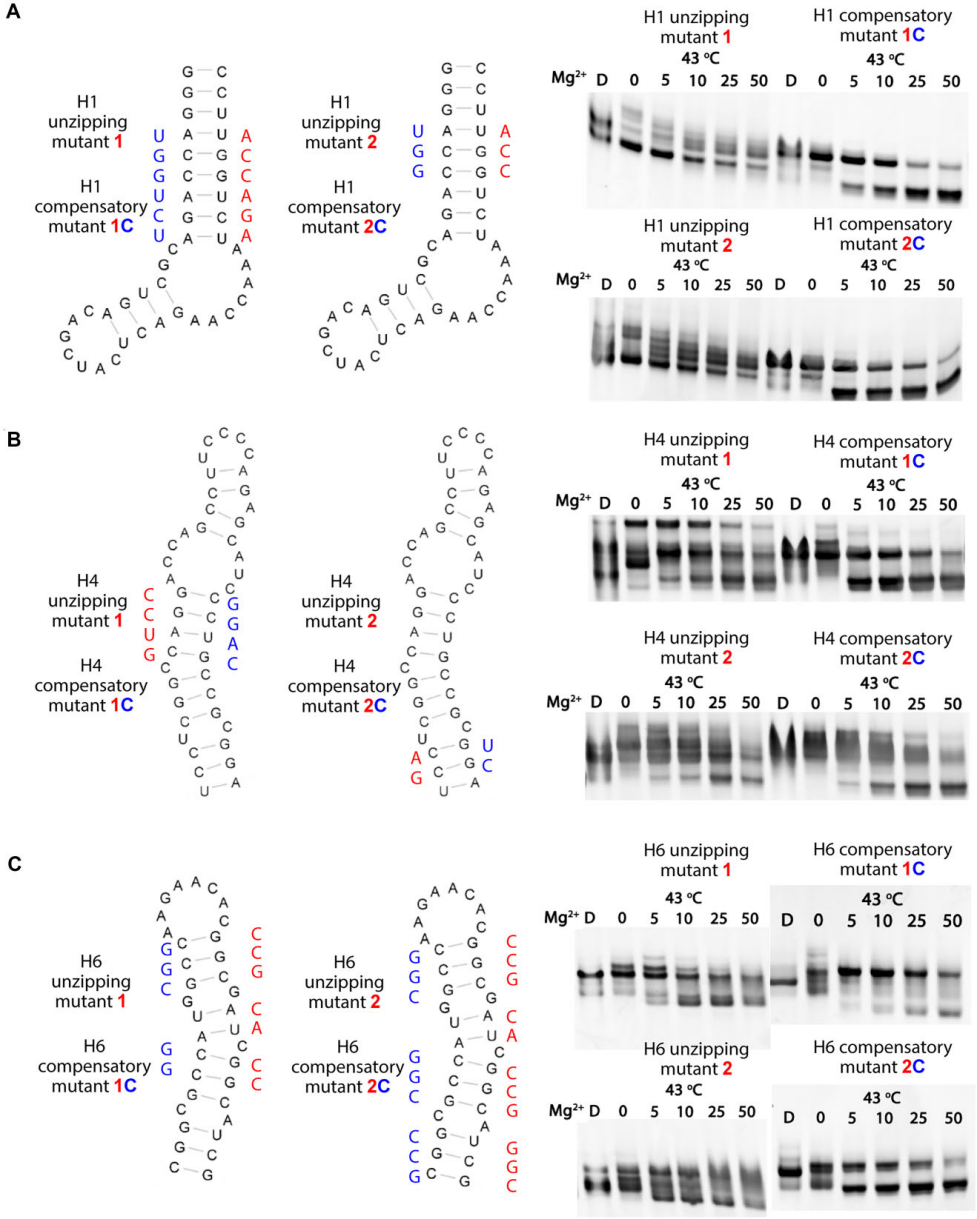
Validation of the predicted secondary structure. Design of unzipping and compensatory mutants based on H1 (**A**), H4 (**B**) and H6 (**C**) stems. The unzipping and corresponding compensatory mutations are rendered. The folding results of these mutants are displayed as described in Figure 1. Unradiolabeled RNAs were used in the folding assay.

To evaluate this experimentally, we introduced long unzipping mutations to disrupt the three stem loops H1, H4 and H6, respectively, and we tested their resulting impact on compaction of the RNA. Based on the observed electrophoretic mobilities of these mutants, the folding capacity of all the unzipping mutants was eliminated or reduced, even at 50 mM Mg^2+^ (Figure 3A–C). To further evaluate the importance of specific stems on UTR26 folding, we introduced the corresponding compensatory mutations to the unzipping mutants, and observed that these restored folding capability (Figure 3A–C). These results are consistent with the predicted structural model and they show that specific RNA stem motifs contribute to the stable folding and architectural form of UTR26 during heat shock.

## Discussion

In this study, we have demonstrated that a specific sequence at the 5′ end of mHsp70 mRNA folds into a compact structure *in vitro*, consisting of both 5′ UTR and upstream ORF sequences. We conducted a comprehensive structural analysis and validation of this structure and determined that 55% of the secondary structure consists of stable stems, formed by sequences spanning the 5′, middle and 3′ regions. Mutational analysis demonstrates that disruption of any of these stems prevents compaction and folding of the molecule. This suggests an interconnected folding mechanism, involving direct interactions between these stems to maintain a stable tertiary structure. Perhaps most significantly, the initiation codon, AUG, is integrated into one of the stems most important for RNA folding, where it becomes an integral part of an intricate, compact structure. This structural arrangement may facilitate the recruitment of ribosomes, either independently or with the assistance of other factors, for cap-independent translation during heat shock. Furthermore, the positioning of the translation initiation site within the core of this structure may cause swift and efficient translation initiation, potentially explaining the molecular mechanism of Hsp70 5′-elements during heat shock.

While this study examined the 5′ UTR in mouse Hsp70 mRNA, it is useful to draw comparison with Hervé Coste’s work, which demonstrated the ability of the human Hsp70 5′ UTR to enhance protein expression (15). However, the 5′ UTR of mouse Hsp70 mRNA differs notably from its human counterpart, thereby limiting the ability to draw parallels between the systems. Nevertheless, it is noteworthy that the 5′ UTR in human Hsp70 mRNA exhibits a high degree of structural complexity. This observation suggests the possibility that the compact 5′ UTR in both human and mouse Hsp70 mRNA may play a positive role in regulating Hsp70 translation. Further investigations would be needed to provide deeper insights into the actual regulatory mechanisms. Similarly, to understand the *in vitro* results presented here, it will be important to conduct future studies that monitor the secondary structure of endogenous Hsp70 mRNA under normal, heat shock and post-heat shock conditions using an *in vivo* chemical probing technique, such as *in vivo* SHAPE-MaP (28,31), enabling a comprehensive functional comparison between *in vitro* and *in vivo* models.

Although these results suggest the existence of a thermally sensitive, compact structure in upstream regions of mHsp70 mRNA, exploring a functional role for this structure will require a meaningful cell-based assay for linking RNA structure in this region with Hsp70 expression during heat shock. In previous studies, a bicistronic assay was used to study the effect of RNA sequence on protein translation, as in studies of IRES elements (10). However, bicistronic assays of Hsp70 function have yielded conflicting results. In some cases, they suggest that the 5′ UTR of Hsp70 mRNA performs IRES or enhancer functions (14,15,32), while in other cases, IRES activity is contraindicated (16,33). Such opposing results on the 5′ UTR of Hsp70 mRNA suggest that traditional bicistronic assays may not be suitable for exploring the influence of Hsp70 mRNA structure on its own translation. For example, the ORF sequence encoded by UTR26 contains several amino acid residues that might influence subsequent translation or folding of the adjacent UTR26-fused reporter gene, thereby affecting the bicistronic assay results. Future studies would benefit from the use of gene editing to create a series of Hsp70 mutant cell lines that enable one to directly monitor RNA structure *in vivo* and the corresponding response of Hsp70 expression to heat shock.

It is possible that post-transcriptional modifications on natural Hsp70 mRNA contribute to the folding transition that we report here. RNA modifications can influence the conformational equilibria of RNA motifs and previous studies have shown that m^6^A modifications on Hsp70 mRNA can regulate cap-independent Hsp70 translation (11–13). The A103 of Hsp70 mRNA was identified as a key m^6^A modification site, as A103C mutation decreases Hsp70 translation dramatically (13). Interestingly, A103 has low SHAPE reactivity and may base pair with U122, which also has low SHAPE reactivity, despite the fact that these nucleotides were not predicted as base-paired in stem H4 (Figure 2B). This suggests the existence of more than one conformation in that position and that disruption of A103:U122 may break the balance in stability of an important motif, destabilizing the whole compact structure. As the predicted model in this study is based on data from the study of unmodified RNA transcripts, it is still an open question how RNA modifications, including m^6^A, modulate riboregulatory motifs and regulate Hsp70 translation.

In summary, biophysical analysis of 5′-terminal sequences within mHsp70 mRNA establishes that upstream portions of this RNA form a compact tertiary structure composed of specific stable stem-loop motifs. Architectural stability of this structure is not solely dependent on sequences from within the 5′ UTR; rather, it requires additional downstream sequences from the ORF region itself, indicating that both coding and noncoding regions of Hsp70 mRNA contribute to riboregulatory regions at the 5′ end of this gene. While future studies are needed to assess the functional role of this folded motif, results reported here establish the compact nature of the mRNA terminus from a gene involved in stress response, suggesting a general mechanism for RNA-based regulation of gene expression.

## Supplementary Material

ugae002_Supplemental_File

## Data Availability

The SHAPE and DMS probing data used in this publication are publicly available for use on Zenodo (https://doi.org/10.5281/zenodo.10474474).

## References

[1] Molto M.D. , Pascual L., de Frutos R. Puff activity after heat shock in two species of the *Drosophila obscura* group. Experientia. 1987; 43:1225–1227. 3691744 10.1007/BF01945535

[2] Craig E.A. , Gross C.A. Is hsp70 the cellular thermometer?. Trends Biochem. Sci. 1991; 16:135–140. 1877088 10.1016/0968-0004(91)90055-z

[3] Radons J. The human HSP70 family of chaperones: where do we stand. Cell Stress Chaperones. 2016; 21:379–404. 26865365 10.1007/s12192-016-0676-6 PMC4837186

[4] Saibil H. Chaperone machines for protein folding, unfolding and disaggregation. Nat. Rev. Mol. Cell Biol. 2013; 14:630–642. 24026055 10.1038/nrm3658 PMC4340576

[5] Sherman M.Y. , Gabai V.L. Hsp70 in cancer: back to the future. Oncogene. 2015; 34:4153–4161. 25347739 10.1038/onc.2014.349 PMC4411196

[6] Joshi S. , Wang T., Araujo T.L.S., Sharma S., Brodsky J.L., Chiosis G. Adapting to stress—chaperome networks in cancer. Nat. Rev. Cancer. 2018; 18:562–575. 29795326 10.1038/s41568-018-0020-9 PMC6108944

[7] Muchowski P.J. , Wacker J.L. Modulation of neurodegeneration by molecular chaperones. Nat. Rev. Neurosci. 2005; 6:11–22. 15611723 10.1038/nrn1587

[8] Vabulas R.M. , Raychaudhuri S., Hayer-Hartl M., Hartl F.U. Protein folding in the cytoplasm and the heat shock response. Cold Spring Harb. Perspect. Biol. 2010; 2:a004390. 21123396 10.1101/cshperspect.a004390 PMC2982175

[9] Gomez-Pastor R. , Burchfiel E.T., Thiele D.J. Regulation of heat shock transcription factors and their roles in physiology and disease. Nat. Rev. Mol. Cell Biol. 2018; 19:4–19. 28852220 10.1038/nrm.2017.73 PMC5794010

[10] Leppek K. , Das R., Barna M. Functional 5′ UTR mRNA structures in eukaryotic translation regulation and how to find them. Nat. Rev. Mol. Cell Biol. 2018; 19:158–174. 29165424 10.1038/nrm.2017.103 PMC5820134

[11] Meyer K.D. , Patil D.P., Zhou J., Zinoviev A., Skabkin M.A., Elemento O., Pestova T.V., Qian S.B., Jaffrey S.R. 5′ UTR m^6^A promotes cap-independent translation. Cell. 2015; 163:999–1010. 26593424 10.1016/j.cell.2015.10.012 PMC4695625

[12] Coots R.A. , Liu X.M., Mao Y., Dong L., Zhou J., Wan J., Zhang X., Qian S.B. m^6^A facilitates eIF4F-independent mRNA translation. Mol. Cell. 2017; 68:504–514. 29107534 10.1016/j.molcel.2017.10.002 PMC5913006

[13] Zhou J. , Wan J., Gao X., Zhang X., Jaffrey S.R., Qian S.B. Dynamic m^6^A mRNA methylation directs translational control of heat shock response. Nature. 2015; 526:591–594. 26458103 10.1038/nature15377 PMC4851248

[14] Rubtsova M.P. , Sizova D.V., Dmitriev S.E., Ivanov D.S., Prassolov V.S., Shatsky I.N. Distinctive properties of the 5′-untranslated region of human hsp70 mRNA. J. Biol. Chem. 2003; 278:22350–22356. 12682055 10.1074/jbc.M303213200

[15] Vivinus S. , Baulande S., van Zanten M., Campbell F., Topley P., Ellis J.H., Dessen P., Coste H. An element within the 5′ untranslated region of human Hsp70 mRNA which acts as a general enhancer of mRNA translation. Eur. J. Biochem. 2001; 268:1908–1917. 11277913 10.1046/j.1432-1327.2001.02064.x

[16] Sun J. , Conn C.S., Han Y., Yeung V., Qian S.B. PI3K-mTORC1 attenuates stress response by inhibiting cap-independent Hsp70 translation. J. Biol. Chem. 2011; 286:6791–6800. 21177857 10.1074/jbc.M110.172882 PMC3057780

[17] Wang W. , Pyle A.M. The RIG-I receptor adopts two different conformations for distinguishing host from viral RNA ligands. Mol. Cell. 2022; 82:4131–4144. 36272408 10.1016/j.molcel.2022.09.029 PMC9707737

[18] Liu T. , Patel S., Pyle A.M. Making RNA: using T7 RNA polymerase to produce high yields of RNA from DNA templates. Methods Enzymol. 2023; 691:185–207. 37914446 10.1016/bs.mie.2023.06.002

[19] Mortimer S.A. , Weeks K.M. A fast-acting reagent for accurate analysis of RNA secondary and tertiary structure by SHAPE chemistry. J. Am. Chem. Soc. 2007; 129:4144–4145. 17367143 10.1021/ja0704028

[20] Somarowthu S. , Legiewicz M., Chillon I., Marcia M., Liu F., Pyle A.M. HOTAIR forms an intricate and modular secondary structure. Mol. Cell. 2015; 58:353–361. 25866246 10.1016/j.molcel.2015.03.006 PMC4406478

[21] Karabiber F. , McGinnis J.L., Favorov O.V., Weeks K.M. QuShape: rapid, accurate, and best-practices quantification of nucleic acid probing information, resolved by capillary electrophoresis. RNA. 2013; 19:63–73. 23188808 10.1261/rna.036327.112 PMC3527727

[22] Rouskin S. , Zubradt M., Washietl S., Kellis M., Weissman J.S. Genome-wide probing of RNA structure reveals active unfolding of mRNA structures *in vivo*. Nature. 2014; 505:701–705. 24336214 10.1038/nature12894 PMC3966492

[23] Low J.T. , Weeks K.M. SHAPE-directed RNA secondary structure prediction. Methods. 2010; 52:150–158. 20554050 10.1016/j.ymeth.2010.06.007 PMC2941709

[24] Liu F. , Somarowthu S., Pyle A.M. Visualizing the secondary and tertiary architectural domains of lncRNA RepA. Nat. Chem. Biol. 2017; 13:282–289. 28068310 10.1038/nchembio.2272 PMC6432788

[25] Mathews D.H. Using an RNA secondary structure partition function to determine confidence in base pairs predicted by free energy minimization. RNA. 2004; 10:1178–1190. 15272118 10.1261/rna.7650904 PMC1370608

[26] Weeks K.M. SHAPE directed discovery of new functions in large RNAs. Acc. Chem. Res. 2021; 54:2502–2517. 33960770 10.1021/acs.accounts.1c00118 PMC8168592

[27] Woodson S.A. , Koculi E. Analysis of RNA folding by native polyacrylamide gel electrophoresis. Methods Enzymol. 2009; 469:189–208. 20946790 10.1016/S0076-6879(09)69009-1 PMC6343852

[28] Siegfried N.A. , Busan S., Rice G.M., Nelson J.A., Weeks K.M. RNA motif discovery by SHAPE and mutational profiling (SHAPE-MaP). Nat. Methods. 2014; 11:959–965. 25028896 10.1038/nmeth.3029 PMC4259394

[29] Zubradt M. , Gupta P., Persad S., Lambowitz A.M., Weissman J.S., Rouskin S. DMS-MaPseq for genome-wide or targeted RNA structure probing *in vivo*. Nat. Methods. 2017; 14:75–82. 27819661 10.1038/nmeth.4057 PMC5508988

[30] Smola M.J. , Rice G.M., Busan S., Siegfried N.A., Weeks K.M. Selective 2′-hydroxyl acylation analyzed by primer extension and mutational profiling (SHAPE-MaP) for direct, versatile and accurate RNA structure analysis. Nat. Protoc. 2015; 10:1643–1669. 26426499 10.1038/nprot.2015.103 PMC4900152

[31] Huston N.C. , Wan H., Strine M.S., de Cesaris Araujo Tavares R., Wilen C.B., Pyle A.M Comprehensive *in vivo* secondary structure of the SARS-CoV-2 genome reveals novel regulatory motifs and mechanisms. Mol. Cell. 2021; 81:584–598. 33444546 10.1016/j.molcel.2020.12.041 PMC7775661

[32] Rocchi L. , Alfieri R.R., Petronini P.G., Montanaro L., Brigotti M. 5′-Untranslated region of heat shock protein 70 mRNA drives translation under hypertonic conditions. Biochem. Biophys. Res. Commun. 2013; 431:321–325. 23291172 10.1016/j.bbrc.2012.12.100

[33] Andreev D.E. , Dmitriev S.E., Terenin I.M., Prassolov V.S., Merrick W.C., Shatsky I.N. Differential contribution of the m^7^G-cap to the 5′ end-dependent translation initiation of mammalian mRNAs. Nucleic Acids Res. 2009; 37:6135–6147. 19696074 10.1093/nar/gkp665 PMC2764426

