# Supplementary material for: A compact regulatory RNA element in mouse Hsp70 mRNA
Source: NAR Mol Med. 2024 Jan 29;1(1):ugae002. doi: 10.1093/narmme/ugae002 (PMC10840451; doi:10.1093/narmme/ugae002)
Supplement: ugae002_Supplemental_File [file ugae002_Supplemental_File.docx]

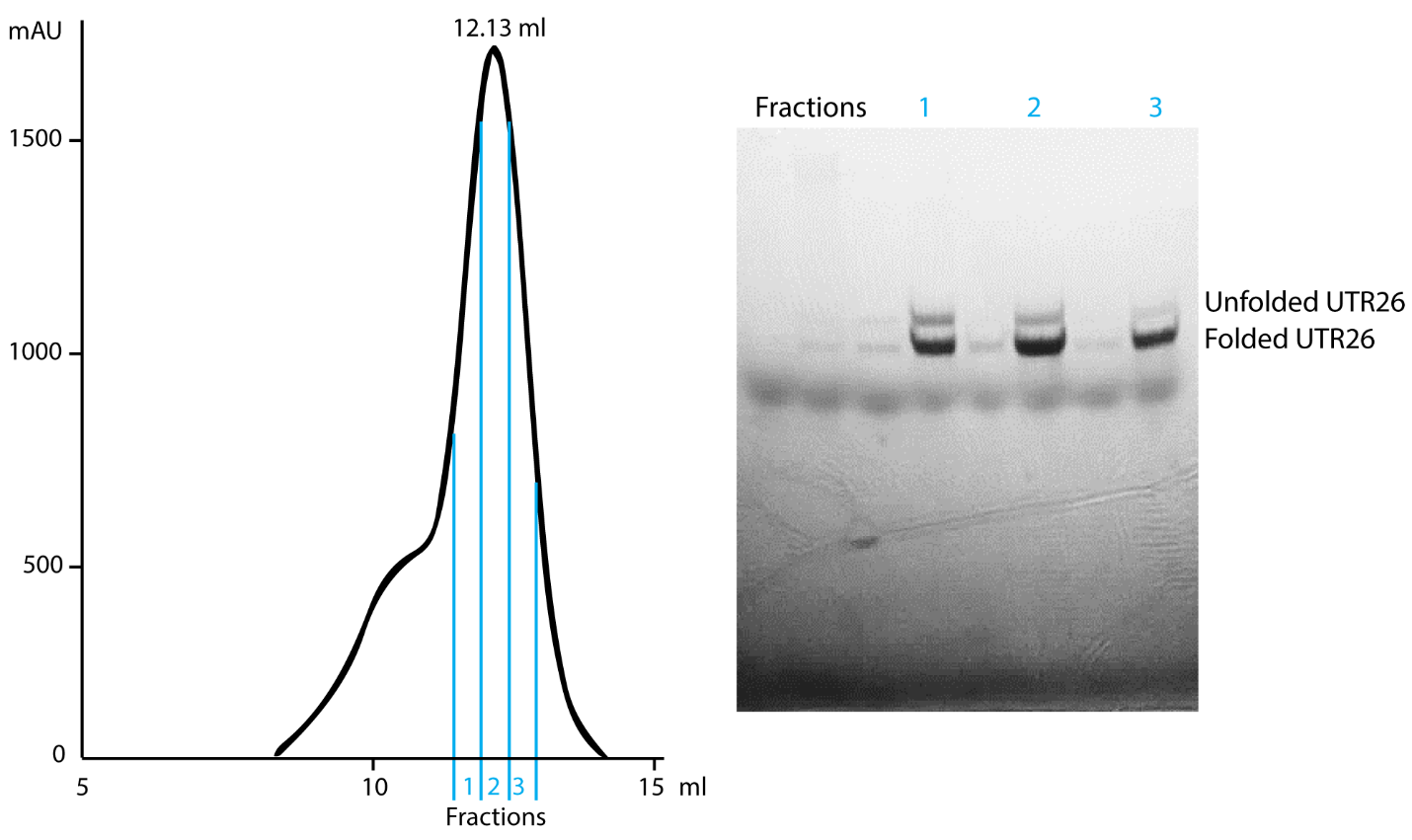


Figure S1 Large scale purification of folded UTR26.

The elution volume of UTR26 after folding from SEC column (24 ml) is rendered. The fraction peak of eluted monomeric folded UTR26 is around 12.13 ml. The eluted UTR26 was assessed using native gel, and folded UTR26 was well separated from unfolded UTR26, especially in the fractions from the tail of the peak. This confirms that the RNA sample (Fraction 3) used for chemical probing is mainly composed of folded UTR26.


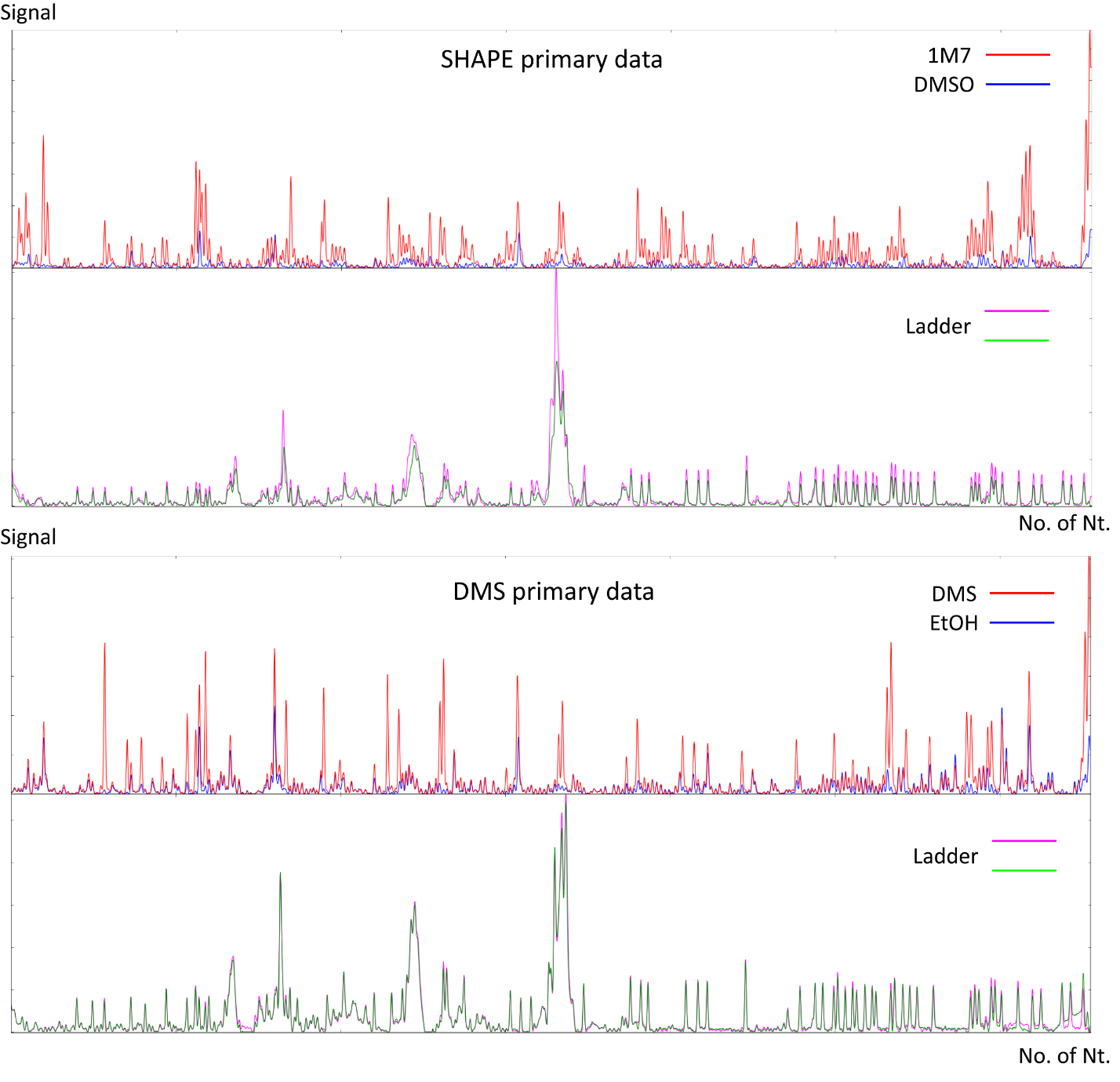


Figure S2 Representative primary data of SHAPE and DMS probing. The primary signal of 1M7/DMS treated (red), DMSO/EtOH treated (blue; negative control), and sequencing ladders (purple and green) in SHAPE and DMS probing experiments are illustrated.

Table S1 DNA sequences of the RNAs used in this study. The canonical start codon is in bold. The unzipping and compensatory mutations are colored in red and blue, respectively.

| Name | Sequence (5' to 3') |
| --- | --- |
| UTR1 (1-231) | ACCAGACGCTGACAGCTACTCAGAACCAAATCTGGTTCCATCCAGAGACAAGCGAAGACAAGAGAAGCAGAGCGAGCGGCGCGTTCCCGATCCTCGGCCAGGACCAGCCTTCCCCAGAGCATCCCTGCCGCGGAGCGCAACCTTCCCAGGAGCATCCCTGCCGCGGAGCGCAACTTTCCCCGGAGCATCCACGCCGCGGAGCGCAGCCTTCCAGAAGCAGAGCGCGGCGCC |
| UTR2 (1-261) | ACCAGACGCTGACAGCTACTCAGAACCAAATCTGGTTCCATCCAGAGACAAGCGAAGACAAGAGAAGCAGAGCGAGCGGCGCGTTCCCGATCCTCGGCCAGGACCAGCCTTCCCCAGAGCATCCCTGCCGCGGAGCGCAACCTTCCCAGGAGCATCCCTGCCGCGGAGCGCAACTTTCCCCGGAGCATCCACGCCGCGGAGCGCAGCCTTCCAGAAGCAGAGCGCGGCGCC**ATG**GCCAAGAACACGGCGATCGGCATCGAC |
| UTR4 (1-285) | ACCAGACGCTGACAGCTACTCAGAACCAAATCTGGTTCCATCCAGAGACAAGCGAAGACAAGAGAAGCAGAGCGAGCGGCGCGTTCCCGATCCTCGGCCAGGACCAGCCTTCCCCAGAGCATCCCTGCCGCGGAGCGCAACCTTCCCAGGAGCATCCCTGCCGCGGAGCGCAACTTTCCCCGGAGCATCCACGCCGCGGAGCGCAGCCTTCCAGAAGCAGAGCGCGGCGCC**ATG**GCCAAGAACACGGCGATCGGCATCGACCTGGGCACCACCTACTCGTGCGTG |
| UTR7 (1-331) | ACCAGACGCTGACAGCTACTCAGAACCAAATCTGGTTCCATCCAGAGACAAGCGAAGACAAGAGAAGCAGAGCGAGCGGCGCGTTCCCGATCCTCGGCCAGGACCAGCCTTCCCCAGAGCATCCCTGCCGCGGAGCGCAACCTTCCCAGGAGCATCCCTGCCGCGGAGCGCAACTTTCCCCGGAGCATCCACGCCGCGGAGCGCAGCCTTCCAGAAGCAGAGCGCGGCGCC**ATG**GCCAAGAACACGGCGATCGGCATCGACCTGGGCACCACCTACTCGTGCGTGGGCGTGTTCCAGCACGGCAAGGTGGAGATCATCGCCAACGACCAGG |
| UTR9 (1-381) | ACCAGACGCTGACAGCTACTCAGAACCAAATCTGGTTCCATCCAGAGACAAGCGAAGACAAGAGAAGCAGAGCGAGCGGCGCGTTCCCGATCCTCGGCCAGGACCAGCCTTCCCCAGAGCATCCCTGCCGCGGAGCGCAACCTTCCCAGGAGCATCCCTGCCGCGGAGCGCAACTTTCCCCGGAGCATCCACGCCGCGGAGCGCAGCCTTCCAGAAGCAGAGCGCGGCGCC**ATG**GCCAAGAACACGGCGATCGGCATCGACCTGGGCACCACCTACTCGTGCGTGGGCGTGTTCCAGCACGGCAAGGTGGAGATCATCGCCAACGACCAGGGCAACCGCACGACCCCCAGCTACGTGGCCTTCACCGACACCGAGCGCCTC |
| UTR10 (1-315) | ACCAGACGCTGACAGCTACTCAGAACCAAATCTGGTTCCATCCAGAGACAAGCGAAGACAAGAGAAGCAGAGCGAGCGGCGCGTTCCCGATCCTCGGCCAGGACCAGCCTTCCCCAGAGCATCCCTGCCGCGGAGCGCAACCTTCCCAGGAGCATCCCTGCCGCGGAGCGCAACTTTCCCCGGAGCATCCACGCCGCGGAGCGCAGCCTTCCAGAAGCAGAGCGCGGCGCC**ATG**GCCAAGAACACGGCGATCGGCATCGACCTGGGCACCACCTACTCGTGCGTGGGCGTGTTCCAGCACGGCAAGGTGGAGATC |
| UTR21 (1-372) | ACCAGACGCTGACAGCTACTCAGAACCAAATCTGGTTCCATCCAGAGACAAGCGAAGACAAGAGAAGCAGAGCGAGCGGCGCGTTCCCGATCCTCGGCCAGGACCAGCCTTCCCCAGAGCATCCCTGCCGCGGAGCGCAACCTTCCCAGGAGCATCCCTGCCGCGGAGCGCAACTTTCCCCGGAGCATCCACGCCGCGGAGCGCAGCCTTCCAGAAGCAGAGCGCGGCGCC**ATG**GCCAAGAACACGGCGATCGGCATCGACCTGGGCACCACCTACTCGTGCGTGGGCGTGTTCCAGCACGGCAAGGTGGAGATCATCGCCAACGACCAGGGCAACCGCACGACCCCCAGCTACGTGGCCTTCACCGACACC |
| UTR22 (1-363) | ACCAGACGCTGACAGCTACTCAGAACCAAATCTGGTTCCATCCAGAGACAAGCGAAGACAAGAGAAGCAGAGCGAGCGGCGCGTTCCCGATCCTCGGCCAGGACCAGCCTTCCCCAGAGCATCCCTGCCGCGGAGCGCAACCTTCCCAGGAGCATCCCTGCCGCGGAGCGCAACTTTCCCCGGAGCATCCACGCCGCGGAGCGCAGCCTTCCAGAAGCAGAGCGCGGCGCC**ATG**GCCAAGAACACGGCGATCGGCATCGACCTGGGCACCACCTACTCGTGCGTGGGCGTGTTCCAGCACGGCAAGGTGGAGATCATCGCCAACGACCAGGGCAACCGCACGACCCCCAGCTACGTGGCCTTC |
| UTR23 (1-354) | ACCAGACGCTGACAGCTACTCAGAACCAAATCTGGTTCCATCCAGAGACAAGCGAAGACAAGAGAAGCAGAGCGAGCGGCGCGTTCCCGATCCTCGGCCAGGACCAGCCTTCCCCAGAGCATCCCTGCCGCGGAGCGCAACCTTCCCAGGAGCATCCCTGCCGCGGAGCGCAACTTTCCCCGGAGCATCCACGCCGCGGAGCGCAGCCTTCCAGAAGCAGAGCGCGGCGCC**ATG**GCCAAGAACACGGCGATCGGCATCGACCTGGGCACCACCTACTCGTGCGTGGGCGTGTTCCAGCACGGCAAGGTGGAGATCATCGCCAACGACCAGGGCAACCGCACGACCCCCAGCTAC |
| UTR24 (1-345) | ACCAGACGCTGACAGCTACTCAGAACCAAATCTGGTTCCATCCAGAGACAAGCGAAGACAAGAGAAGCAGAGCGAGCGGCGCGTTCCCGATCCTCGGCCAGGACCAGCCTTCCCCAGAGCATCCCTGCCGCGGAGCGCAACCTTCCCAGGAGCATCCCTGCCGCGGAGCGCAACTTTCCCCGGAGCATCCACGCCGCGGAGCGCAGCCTTCCAGAAGCAGAGCGCGGCGCC**ATG**GCCAAGAACACGGCGATCGGCATCGACCTGGGCACCACCTACTCGTGCGTGGGCGTGTTCCAGCACGGCAAGGTGGAGATCATCGCCAACGACCAGGGCAACCGCACGACC |
| UTR25 (1-336) | ACCAGACGCTGACAGCTACTCAGAACCAAATCTGGTTCCATCCAGAGACAAGCGAAGACAAGAGAAGCAGAGCGAGCGGCGCGTTCCCGATCCTCGGCCAGGACCAGCCTTCCCCAGAGCATCCCTGCCGCGGAGCGCAACCTTCCCAGGAGCATCCCTGCCGCGGAGCGCAACTTTCCCCGGAGCATCCACGCCGCGGAGCGCAGCCTTCCAGAAGCAGAGCGCGGCGCC**ATG**GCCAAGAACACGGCGATCGGCATCGACCTGGGCACCACCTACTCGTGCGTGGGCGTGTTCCAGCACGGCAAGGTGGAGATCATCGCCAACGACCAGGGCAAC |
| UTR26 (1-327) | ACCAGACGCTGACAGCTACTCAGAACCAAATCTGGTTCCATCCAGAGACAAGCGAAGACAAGAGAAGCAGAGCGAGCGGCGCGTTCCCGATCCTCGGCCAGGACCAGCCTTCCCCAGAGCATCCCTGCCGCGGAGCGCAACCTTCCCAGGAGCATCCCTGCCGCGGAGCGCAACTTTCCCCGGAGCATCCACGCCGCGGAGCGCAGCCTTCCAGAAGCAGAGCGCGGCGCC**ATG**GCCAAGAACACGGCGATCGGCATCGACCTGGGCACCACCTACTCGTGCGTGGGCGTGTTCCAGCACGGCAAGGTGGAGATCATCGCCAACGAC |
| H1 unzipping mutant 1 | ACCAGACGCTGACAGCTACTCAGAACCAAAAGACCATCCATCCAGAGACAAGCGAAGACAAGAGAAGCAGAGCGAGCGGCGCGTTCCCGATCCTCGGCCAGGACCAGCCTTCCCCAGAGCATCCCTGCCGCGGAGCGCAACCTTCCCAGGAGCATCCCTGCCGCGGAGCGCAACTTTCCCCGGAGCATCCACGCCGCGGAGCGCAGCCTTCCAGAAGCAGAGCGCGGCGCC**ATG**GCCAAGAACACGGCGATCGGCATCGACCTGGGCACCACCTACTCGTGCGTGGGCGTGTTCCAGCACGGCAAGGTGGAGATCATCGCCAACGAC |
| H1 compensatory mutant 1C | TGGTCTCGCTGACAGCTACTCAGAACCAAAAGACCATCCATCCAGAGACAAGCGAAGACAAGAGAAGCAGAGCGAGCGGCGCGTTCCCGATCCTCGGCCAGGACCAGCCTTCCCCAGAGCATCCCTGCCGCGGAGCGCAACCTTCCCAGGAGCATCCCTGCCGCGGAGCGCAACTTTCCCCGGAGCATCCACGCCGCGGAGCGCAGCCTTCCAGAAGCAGAGCGCGGCGCC**ATG**GCCAAGAACACGGCGATCGGCATCGACCTGGGCACCACCTACTCGTGCGTGGGCGTGTTCCAGCACGGCAAGGTGGAGATCATCGCCAACGAC |
| H1 unzipping mutant 2 | ACCAGACGCTGACAGCTACTCAGAACCAAATCTCCATCCATCCAGAGACAAGCGAAGACAAGAGAAGCAGAGCGAGCGGCGCGTTCCCGATCCTCGGCCAGGACCAGCCTTCCCCAGAGCATCCCTGCCGCGGAGCGCAACCTTCCCAGGAGCATCCCTGCCGCGGAGCGCAACTTTCCCCGGAGCATCCACGCCGCGGAGCGCAGCCTTCCAGAAGCAGAGCGCGGCGCC**ATG**GCCAAGAACACGGCGATCGGCATCGACCTGGGCACCACCTACTCGTGCGTGGGCGTGTTCCAGCACGGCAAGGTGGAGATCATCGCCAACGAC |
| H1 compensatory mutant 2C | TGGAGACGCTGACAGCTACTCAGAACCAAATCTCCATCCATCCAGAGACAAGCGAAGACAAGAGAAGCAGAGCGAGCGGCGCGTTCCCGATCCTCGGCCAGGACCAGCCTTCCCCAGAGCATCCCTGCCGCGGAGCGCAACCTTCCCAGGAGCATCCCTGCCGCGGAGCGCAACTTTCCCCGGAGCATCCACGCCGCGGAGCGCAGCCTTCCAGAAGCAGAGCGCGGCGCC**ATG**GCCAAGAACACGGCGATCGGCATCGACCTGGGCACCACCTACTCGTGCGTGGGCGTGTTCCAGCACGGCAAGGTGGAGATCATCGCCAACGAC |
| H4 unzipping mutant 1 | ACCAGACGCTGACAGCTACTCAGAACCAAATCTGGTTCCATCCAGAGACAAGCGAAGACAAGAGAAGCAGAGCGAGCGGCGCGTTCCCGATCCTCGGCGTCCACCAGCCTTCCCCAGAGCATCCCTGCCGCGGAGCGCAACCTTCCCAGGAGCATCCCTGCCGCGGAGCGCAACTTTCCCCGGAGCATCCACGCCGCGGAGCGCAGCCTTCCAGAAGCAGAGCGCGGCGCC**ATG**GCCAAGAACACGGCGATCGGCATCGACCTGGGCACCACCTACTCGTGCGTGGGCGTGTTCCAGCACGGCAAGGTGGAGATCATCGCCAACGAC |
| H4 compensatory mutant 1C | ACCAGACGCTGACAGCTACTCAGAACCAAATCTGGTTCCATCCAGAGACAAGCGAAGACAAGAGAAGCAGAGCGAGCGGCGCGTTCCCGATCCTCGGCGTCCACCAGCCTTCCCCAGAGCATCGGACCCGCGGAGCGCAACCTTCCCAGGAGCATCCCTGCCGCGGAGCGCAACTTTCCCCGGAGCATCCACGCCGCGGAGCGCAGCCTTCCAGAAGCAGAGCGCGGCGCC**ATG**GCCAAGAACACGGCGATCGGCATCGACCTGGGCACCACCTACTCGTGCGTGGGCGTGTTCCAGCACGGCAAGGTGGAGATCATCGCCAACGAC |
| H4 unzipping mutant 2 | ACCAGACGCTGACAGCTACTCAGAACCAAATCTGGTTCCATCCAGAGACAAGCGAAGACAAGAGAAGCAGAGCGAGCGGCGCGTTCCCGATGATCGGCCAGGACCAGCCTTCCCCAGAGCATCCCTGCCGCGGAGCGCAACCTTCCCAGGAGCATCCCTGCCGCGGAGCGCAACTTTCCCCGGAGCATCCACGCCGCGGAGCGCAGCCTTCCAGAAGCAGAGCGCGGCGCC**ATG**GCCAAGAACACGGCGATCGGCATCGACCTGGGCACCACCTACTCGTGCGTGGGCGTGTTCCAGCACGGCAAGGTGGAGATCATCGCCAACGAC |
| H4 compensatory mutant 2C | ACCAGACGCTGACAGCTACTCAGAACCAAATCTGGTTCCATCCAGAGACAAGCGAAGACAAGAGAAGCAGAGCGAGCGGCGCGTTCCCGATGATCGGCCAGGACCAGCCTTCCCCAGAGCATCCCTGCCGCTCAGCGCAACCTTCCCAGGAGCATCCCTGCCGCGGAGCGCAACTTTCCCCGGAGCATCCACGCCGCGGAGCGCAGCCTTCCAGAAGCAGAGCGCGGCGCC**ATG**GCCAAGAACACGGCGATCGGCATCGACCTGGGCACCACCTACTCGTGCGTGGGCGTGTTCCAGCACGGCAAGGTGGAGATCATCGCCAACGAC |
| H6 unzipping mutant 1 | ACCAGACGCTGACAGCTACTCAGAACCAAATCTGGTTCCATCCAGAGACAAGCGAAGACAAGAGAAGCAGAGCGAGCGGCGCGTTCCCGATCCTCGGCCAGGACCAGCCTTCCCCAGAGCATCCCTGCCGCGGAGCGCAACCTTCCCAGGAGCATCCCTGCCGCGGAGCGCAACTTTCCCCGGAGCATCCACGCCGCGGAGCGCAGCCTTCCAGAAGCAGAGCGCGGCGCC**ATG**GCCAAGAACACCCGGCACCCCATCGACCTGGGCACCACCTACTCGTGCGTGGGCGTGTTCCAGCACGGCAAGGTGGAGATCATCGCCAACGAC |
| H6 compensatory mutant 1C | ACCAGACGCTGACAGCTACTCAGAACCAAATCTGGTTCCATCCAGAGACAAGCGAAGACAAGAGAAGCAGAGCGAGCGGCGCGTTCCCGATCCTCGGCCAGGACCAGCCTTCCCCAGAGCATCCCTGCCGCGGAGCGCAACCTTCCCAGGAGCATCCCTGCCGCGGAGCGCAACTTTCCCCGGAGCATCCACGCCGCGGAGCGCAGCCTTCCAGAAGCAGAGCGCGGCGGG**ATG**CGGAAGAACACCCGGCACCCCATCGACCTGGGCACCACCTACTCGTGCGTGGGCGTGTTCCAGCACGGCAAGGTGGAGATCATCGCCAACGAC |
| H6 unzipping mutant 2 | ACCAGACGCTGACAGCTACTCAGAACCAAATCTGGTTCCATCCAGAGACAAGCGAAGACAAGAGAAGCAGAGCGAGCGGCGCGTTCCCGATCCTCGGCCAGGACCAGCCTTCCCCAGAGCATCCCTGCCGCGGAGCGCAACCTTCCCAGGAGCATCCCTGCCGCGGAGCGCAACTTTCCCCGGAGCATCCACGCCGCGGAGCGCAGCCTTCCAGAAGCAGAGCGCGGCGCC**ATG**GCCAAGAACACCCGGCACCCGAGGCACCTGGGCACCACCTACTCGTGCGTGGGCGTGTTCCAGCACGGCAAGGTGGAGATCATCGCCAACGAC |
| H6 compensatory mutant 2C | ACCAGACGCTGACAGCTACTCAGAACCAAATCTGGTTCCATCCAGAGACAAGCGAAGACAAGAGAAGCAGAGCGAGCGGCGCGTTCCCGATCCTCGGCCAGGACCAGCCTTCCCCAGAGCATCCCTGCCGCGGAGCGCAACCTTCCCAGGAGCATCCCTGCCGCGGAGCGCAACTTTCCCCGGAGCATCCACGCCGCGGAGCGCAGCCTTCCAGAAGCAGAGCGGCCCCGG**ATG**CGGAAGAACACCCGGCACCCGAGGCACCTGGGCACCACCTACTCGTGCGTGGGCGTGTTCCAGCACGGCAAGGTGGAGATCATCGCCAACGAC |
